# Influence of a Structured Teaching on Targeted Pelvic Floor Muscle Contraction Ability in Pregnant Women: The pelviTrust Trial

**DOI:** 10.3390/healthcare14050651

**Published:** 2026-03-04

**Authors:** Konstanze Weinert, Ulrike Keim, Anna-Lena Wawers, Nina Gärtner-Tschacher, Claudia F. Plappert

**Affiliations:** 1 Institute for Health Science, Section of Midwifery Science, University Hospital Tuebingen, 72076 Tuebingen, Germany; ulrike.keim@med.uni-tuebingen.de (U.K.); anna-lena.wawers@student.uni-tuebingen.de (A.-L.W.); claudia.plappert@med.uni-tuebingen.de (C.F.P.); 2 Section of Physiotherapy, University Hospital Tuebingen, 72076 Tuebingen, Germany; nina.gaertner-tschacher@med.uni-tuebingen.de

**Keywords:** pregnancy, pregnant women, pelvic floor, pelvic floor dysfunctions, midwifery care, teaching, education

## Abstract

**Background**: Pelvic floor muscle dysfunction (PFD) is common during pregnancy. To counteract pregnancy-associated PFD, women require sufficient knowledge and structured guidance on correct pelvic floor muscle (PFM) contraction to improve PFM perception and functional control. Identifying pregnant women who are unable to perform correct PFM contraction despite structured teaching may allow early referral for rehabilitative measures. **Objective:** At measurement stage 1, this study aims to investigate the influence of structured PFM teaching on pregnant women’s ability to perform targeted PFM contraction (tPFMC-A), assess PFM strength, and describe possible early PFD symptoms. **Material and Methods**: “pelviTrust” is a two-arm randomized, controlled longitudinal study and has been conducted in the Department of Midwifery Science, University of Tuebingen since February 2023. The study sample comprised 221 healthy pregnant women with singleton pregnancy at 18–22 weeks of gestation. The intervention group (IG; *n* = 113) (69 nulliparous, 40 primiparous and four biparous) completed the validated German Pelvic Floor Questionnaire for Pregnant and Postpartum Women (*GPFQppw*) and received individualized midwife-led teaching on PFM anatomy, functional activation and PFM-friendly behaviour, followed by visual inspection and vaginal palpation. Objective-targeted PFMC ability (tPFMC-A) and PFM strength (modified Oxford Scale) were compared with self-assessed ability. The control group (*n* = 101) (61 nulliparous, 38 primiparous, and two biparous) receives routine prenatal and postnatal care and completes the *GPFQppw*. The present analysis focuses exclusively on the IG at T1. **Results**: At T1, 88% of the 113 women in the IG believed they could contract their PFM, but only 68% demonstrated a correct tPFMC-A on visual inspection. Following structured teaching with individualized feedback, 97% achieved correct PFM contraction while 2.7% still had deficits. The median PFM strength was three on the modified Oxford Scale (interquartile range: 3–4). Stress urinary incontinence and flatulence were the most frequently reported symptoms. Primiparous and multiparous women reported urinary incontinence and descensus symptoms more often than nulliparous women (*p* < 0.001). **Conclusions**: At the first prenatal assessment, pregnant women often overestimate their ability to contract their PFM correctly. A structured, midwife-led PFM teaching improves objectively assessed PFM contraction ability and may be integrated into routine antenatal care to support PFM health in pregnant women.

## 1. Introduction

Women are at high risk of pelvic floor dysfunction (PFD) in connection with pregnancy and childbirth. Possible symptoms include various forms of urinary incontinence, anal incontinence such as fecal incontinence and/or flatulence, descensus problems and sexual disorders [1,2]. Hübner et al. (2022) reported in their review that the prevalence of urinary incontinence during pregnancy is up to 58% [3]. Immediately postpartum, up to 36% of women are affected by urinary incontinence and 8% by fecal incontinence [3]. MacArthur et al. (2011) and Jelovsek et al. (2018) demonstrated in their long-term studies that women with postpartum urinary incontinence in early postpartum have a significantly increased risk of developing urinary incontinence [4,5]. The main risk factors for developing PFD in the context of pregnancy and childbirth are the physiological (hormonal) changes associated with pregnancy itself, which in themselves lead to changes in muscles and connective tissue and to a descent of the female pelvic organs [6]. In addition, existing obesity [7], previous births [6,7,8], especially with previous vaginal-operative births [1,8], and severe obstetric injuries (OASIs) [9] are risk factors that promote PDF. Overall, PFDs are a shameful topic for affected women, associated with high physical and psychological stress and reduced quality of life [10,11].

The benefits of rehabilitative PFM training for women with pre-existing PFD are the subject of controversy. Even though Dumoulin et al. (2018) conclude in their Cochrane Review, which included 31 studies with 1817 women from 14 countries, that PFM training can cure or improve the various symptoms of urinary incontinence, further in-depth research is needed to measure long-term success [12]. Woodley et al. (2020) showed in their Cochrane Review of 46 trials with 10,832 pregnant participants from 21 countries that there was no positive effect for effective PFM training in pregnant women with pre-existing PFD [13]. Current studies show that women with a single pregnancy during their first pregnancy, in particular, benefit from preventive PFM training: Various randomized controlled trials, including those by Mørkved et al. (2003), Stafne et al. (2012), Pelaez et al. (2014), Sangsawang and Sangsawang (2016) and Jinapun and Sangnucktham (2024), have demonstrated significant reductions in urinary incontinence among pregnant women who undertook structured antenatal PFM training [14,15,16,17,18]. Although these trials differ in sample size and protocol, they consistently report positive effects [14,15,16,17,18]. Structured PFM training during pregnancy has also been associated with reduced postpartum urinary incontinence. Reilly et al. (2002), Mørkved et al. (2003) and Stafne et al. (2012) reported significantly fewer symptoms among women who received antenatal PFM training, even at three months postpartum [14,15,19]. In contrast to the positive evidence cited for the prevention of urinary incontinence through antenatal PFM training, various reviews indicate that there is a lack of reliable data on the prevention of fecal incontinence [13,20,21].

Against this background, various international guidelines recommend preventive or rehabilitative PFM training for women, especially pregnant women [22,23,24]. Effective and targeted PFM training requires women to be able to tense their PFM in a targeted and correct manner [22,24]. However, various studies show that a significant proportion of women of all ages is unable to perform correct PFM contraction [25,26,27]. The authors postulate that a lack of PFM control and proprioception can be the basis for incorrect PFM contraction and that these must first be practised and supported sensitively and under adequate guidance in order to achieve any improvement in tension behaviour. The authors further point out that in cases of severe PFM deficits and/or pre-existing PFD, correct PFM contraction may not be possible [24,25,26].

It is also known that women of reproductive age have knowledge deficits regarding PFDs and their prevention approaches [28,29,30]. This knowledge deficit also appears to be entrenched in women in the context of pregnancy and childbirth: in their recent review, Weinert and Plappert (2025) highlighted existing gaps in education and knowledge among pregnant women regarding the anatomy, function and dysfunction of the PFM and the prevention of PFDs [31]. It was also shown that pregnant women with various parities lack knowledge and feel uncertain about their ability to tense their PFM in a targeted manner [31]. Another barrier to promoting PFM health among pregnant women in Germany is posed by the current maternity guidelines [32]. These do not provide for prenatal risk assessment of PFM health in the maternity record. Similarly, the ability of a pregnant women to adequately tense and relax her PFM is not assessed on this stage [33].

In Germany, midwives care for and support women during pregnancy, childbirth and the postnatal period. They accompany women through pregnancy, birth and postpartum period and are legally mandated to provide antenatal classes, birth preparation, postpartum care and specific PFM training [34,35]. The German national health objective “Health around childbirth” underscores that midwives should promote physiological processes and empower women to care for their own health [36]. In practice, midwives teach pregnant women about PFM anatomy, the impact of hormonal and mechanical changes, and PFM-friendly behaviour during daily activities; in the early puerperium, they assess PFM function, support correct activation and relaxation and initiate rehabilitative exercises [35,37]. Should functional deficits or symptoms of PFD be detected, they refer women to physiotherapists or physicians. Thus, midwives combine counselling, physical examination, supervised exercise instruction and referral management to support PFM health and provide comprehensive guidance on preventing and managing PFDs.

This background shows that the midwifery profession plays an essential role in promoting women’s pelvic floor health. The inclusion of the midwifery science perspective also underscores this relevance: pregnant women need targeted teaching, i.e., adequate knowledge transfer about PFM, pregnancy-specific changes and possible preventive measures such as PFM training. Consciously learning about the PFM region, understanding how it works and becoming aware of one’s own PFM are ideal preparation for effective PFM training. Effective functional training should be preceded by verification of correct PFM contraction through visual inspection, instruction and verbal feedback. In addition, the simple but proven method of vaginal palpation according to the Oxford Grading Scale by Laycock and Jerwood [38] enables a differentiated assessment of muscle strength and thus conclusions about existing or developing dysfunctions [23,24].

Based on these relevant findings, we designed the prospective randomized controlled trial (RCT) “pelviTrust” as a longitudinal two-arm study to investigate whether structured PFM teaching and PFM training during pregnancy improve objective PFM function and women’s subjective perception of PFM health from early pregnancy through the postpartum period. The intervention group (IG) receives midwife-led teaching (instruction, inspection, feedback and vaginal palpation) plus structured PFM training, whereas the control group (CG) receives routine prenatal and postnatal care and completes the validated German Pelvic Floor Questionnaire for Pregnant and Postpartum Women (*GPFQppw*) [39,40]. Data collection takes place at three defined points: in the 18th–22nd week of pregnancy (T1), 6–8 weeks postpartum (T2) and 9 months postpartum (T3). There were no accompanying treatments for any of the participants that were prohibited during the entire study.

This article reports only the first measurement point (T1) and analysis data from the intervention group. This focus allows for a detailed examination of PFM function and perception at the early pregnancy. The aim of this article is to explore pregnant women’s subjective perception of their ability to perform targeted PFM contractions and to contrast this perception with objectively assessed contraction ability obtained through standardized functional diagnostics. In addition, PFM strength and early symptoms of PFD are described to provide a comprehensive picture of functional and perceived PFM health at T1.

Against this background, three different research questions can be derived: How do pregnant women subjectively assess their ability to perform targeted pelvic floor muscle contractions (t-PFMC-A), and how does this correspond to objectively assessed contraction ability? To what extent does structured midwife-led PFM teaching support correct and targeted PFM activation at this early stage of pregnancy? Which levels of PFM strength and which symptoms of PFD are already present at T1 in the intervention group?

## 2. Materials and Methods

### 2.1. Design and Ethical Approval

The prospective, randomized controlled two-arm intervention study “pelviTrust” has been conducted since 2023 at the Department of Midwifery Science, University of Tuebingen. The study was approved by the Ethics Committee of the Medical Faculty of the University of Tuebingen (150/2022BO2). It is listed on the World Health Organization’s International Platform for Clinical Trials and in the German Clinical Trials Register (DRKS) under registration number DRKS00030965 and follows the Spirit 2013 Statement according to Chan et al. [41] based on the CONSORT Statement (2010) [42].

### 2.2. Sample Size and Recruitment

The total sample size was based on calculations using the statistical power analysis tool G*Power 3.1 [43]. An a priori power analysis was performed in G*Power 3.1 for unpaired samples test (two-sided, α = 0.05, power = 0.80) using a medium, conservative effect size (Cohen’s d ≈ 4.0) based on previous studies on PFM health [44]. A total sample size of *n* = 200 was calculated. Given the longitudinal design of the study, a dropout rate of 20% was assumed [19,45,46], which resulted in a targeted recruitment of *n* = 120 participants per study arm.

Following the study protocol, participants were recruited between the 18th and 22nd week of pregnancy by registered gynecologists and freelance midwives from the Tuebingen/Reutlingen area and from the prenatal diagnostics outpatient clinic at Tuebingen University Hospital. Inclusion criteria are as follows: healthy singleton pregnancy, age ≥18 years, 18th–22nd week of pregnancy, sufficient knowledge of German. Primary exclusion criteria: placenta praevia, risk of premature birth. Secondary exclusion criteria are as follows: immediate need for treatment based on the German Pelvic Floor Questionnaire for pregnant and postpartum women (*GPFQppw*), defined as inability to voluntarily contract the PFM and/or an assessment of lack of physiological PFM strength [38]. Participants were enrolled in the study by two individuals—the midwife who conducted the study and the intervention, and the associated principal investigator. The eligible participants were randomly allocated to the intervention Group (IG) or control group (CG). Urn randomization with “putting back” was chosen as the randomization method [47,48]. Randomisation was performed by one person, which is the midwife conducting the study. All interventions and outcome assessments were likewise performed by this midwife, adhering to clear criteria. Participants meeting secondary exclusion criteria were referred for appropriate physiotherapeutic or medical treatment. Blinding of participants was not possible due to the study design.

Our study is a pilot study. Although the study is conducted according to the study protocol, the randomization procedure, lack of blinding and the implementation and evaluation of the intervention had the potential for selection, performance and/or detection bias and could therefore affect the internal validity of the study. This context is reflected in the chapter on limitations.

### 2.3. Study Focus and Outcomes

The present analysis focuses exclusively on the first measurement point (T1) during early pregnancy and includes data from participants of the intervention group only. The primary outcome was the comparison of self-assessed and objectively evaluated targeted tPFMC-A. Secondary outcomes comprised PFM strength assessed by the modified Oxford Grading Scale and the prevalence of self-reported PFD symptoms.

### 2.4. Statistical Analysis

Numerical demographic and clinical data were presented as mean ± SD or median (IQR) depending on the distribution of the data, where categorical variables were absolute and relative frequencies. Categorical data were tested using the chi-square test, and numerical data were tested using the *t*-test. A *p*-value ≤ 0.05 was considered significant. Analyses were performed using IBM SPSS Statistics, version 30.0.

### 2.5. Intervention and Assessments

The following section describes the assessment procedure for the self-assessed and reported PFM health of the study participants and the objective functional diagnostic procedures and assessment criteria of the “pelviTrust” study. These were performed exclusively by the same midwife at time T1 to ensure consistent application of the protocol and reduce inter-rater variability.

(I) Self-assessed targeted pelvic floor contraction ability (tPFMC-A) and pelvic floor health

The *GPFQppw* [40] was used to represent self-reported PFM health, which also integrates the assessment of self-assessed tPFMC-A, and was sent to all study participants at T1. The UNIPARK^®^ (TIVIAN) survey software, version 3, was used for this purpose [49]. The *GPFQppw* consists of a total of six sub-areas and initially covers the participants’ existing basic risks: the first domain records birth- and risk-related data such as age, body mass index (BMI), nicotine abuse, anamnestic PFD, and self-assessed PFM contraction ability. The response options relating to medical history and PFM-contracting ability are “no”/“I don’t know”/“yes”. A total of 41 other items relating to symptoms and functional limitations is recorded in four additional areas: bladder function (16 items), bowel function (11 items), descent symptoms (five items) and sexuality (nine items). Responses are given on a four-point scale from 0 (‘never’) to 3 (“always”/“most of the time”/“daily”). Area-specific functional limitations (items) were assigned values (0–3) and calculated. Higher values indicate greater symptom severity and greater functional impairment and risk exposure. The final sixth part of the *GPFQppw* collects data on parity, for previous births, type of delivery, birth weight of the child and severity of birth injuries (OASIs). All items from the six different domains were evaluated in a topic-specific manner and are compared in Table 1, Table 2, Table 3 and Table 4 of this article. The survey was expanded by the research team to include questions on socio-demographic background.

The chosen approach to the exclusion criteria corresponds to a pragmatic approach that aims to realistically reflect PFM health based on a broader, less selected population. The following parameters were defined as requiring immediate treatment according to *GPFQppw:* inability to contract the PFM (answer “no”); and/or assessment vaginal palpation: lack of physiological PFM strength [38] at T1.

(II) Structured PFM teaching
(IIa) Knowledge transfer and guidance on correct PFM contraction

Participants received a comprehensive introduction to the anatomy and function of the PFM, supported by models and illustrations. Self-palpation of bony pelvic structures served to promote physical and haptic understanding. Breathing exercises promoted awareness of diaphragmatic and PFM movement. In addition, measures for the prevention of pregnancy-associated PFDs were taught, e.g., PFM-friendly behaviour in everyday life (voluntary pre-contraction, “The Knack” when lifting, sneezing, coughing) [50,51,52] and economical movement patterns (bending, lifting, carrying, standing up, toilet behaviour) [53,54]. Finally, verbal instructions were given for a PFM contraction exercise (sitting or lying down), based on Bump et al. (1991) [25], Mørkved and Bø et al. (2015) and Mørkved (2024) [55,56], with a focus on breathing and maximum, isolated PFM contraction, followed by relaxation of the PFM.

(IIb) Visual inspection and feedback

For the subsequent objective examination of PFM contraction, the participants performed a maximum voluntary PFM contraction in a standardized supine position (35° headrest, legs bent, bladder emptied). This was followed by a visual inspection of the tPFMC-A. Contraction, relaxation and co-contractions were evaluated according to the criteria of the International Continence Society (ICS) (2021) [57].

A correct, voluntary maximum PFM contraction causes the bulbospongiosus/bulbocavernosus, ischiocavernosus and transversus perinei muscles to contract inwards and upwards. This results in a ventrocephalic movement of the vulva, perineum and anus. The objective assessment was performed according to ICS criteria: present/uncertain/absent/straining. The subsequent relaxation of the PFM is assessed as present/partially present or absent [57]. In addition, cough tests and pushing tests were performed at this stage to assess involuntary reflex contraction and to screen for possible descent symptoms [57].

It was observed whether the PFM contraction could be performed in isolation or whether co-contractions, such as tensing the hip adductors, gluteal or abdominal muscles, prevented or influenced the correct PFM contraction [57]. In addition, it was assessed whether respiratory flow, as holding one’s breath, also leads to reduced PFM contraction [58].

Each participant received verbal feedback on the correctness of their PFM contraction. In cases of missing or uncertain PFM contraction, co-contractions or disturbed breathing flow, individualized and differentiated verbal guidance was provided to improve physiological contraction behaviour [25,26,27,57]. Guidance on safe and correct PFM contraction was provided using the following instructions based on Mørkved and Bø et al. (2015) and Mørkved (2024) [55,56]: “With your next exhalation, squeeze your anal sphincter, vagina and urethra as powerfully as possible inwards and upwards. The more powerfully you squeeze your PFM inwards and upwards, the closer your sitting bones will come together, and your lower abdominal muscles will tense only slightly. Imagine stopping the flow of urine when urinating and preventing bowel movements and flatulence. Hold this tension for 8 s. Breathe in and out slowly and regularly, maintaining a relaxed breathing rhythm.”

To relax the PFM, the following verbal instruction was given: “Now slowly relax your pelvic floor. Slowly release your tension from the inside-up to the inside-down. The urethra, vagina and anal sphincter are noticeably relaxed again, one after the other.” In cases of co-contractions, the instructions listed above were repeated. Co-contracting muscle groups were explicitly named and pointed out so that adequate and exclusive PFM contraction could be addressed. Specific breathing instructions emphasized conscious inhalation and exhalation while simultaneously perceiving PFM activity. PFM contraction was consciously initiated during exhalation to utilize the synergistic effects of diaphragmatic relaxation with simultaneous concentric PFM activation. Continued breathing with further tensioning of the PFM should be ensured [58].

(III) Objective functional diagnostics: vaginal palpation and determination of PFM strength (MOS)

This was followed by vaginal palpation to assess individual PFM strength. This was performed with an examination finger [57,59] and in the standardized supine position described above. The participants were asked to perform a voluntary maximum PFM contraction as described. The following parameters from the ICS (2021) were used for the objective evaluation of the results [57]: Vaginal palpation measures individual PFM strength during voluntary PFM contraction, which is assessed using the six-point modified Oxford Grading Scale (MOS) according to Laycock and Jerwood (2001) [38]: 0 = no contraction; 1 = twitching of individual muscle fibres or slight fluttering; 2 = weak contraction, no lift of the examining finger; 3 = good contraction, a lift of the examining finger is noticeable; 4 = moderate contraction, examining finger is lifted against slight resistance; 5 = strong contraction, lifting the examining finger against strong resistance. The examination was performed using the index finger. This was placed approximately 4 cm into the vagina and positioned at approximately 5 and 7 o’clock with moderate pressure on the PFM [38,57].

After the voluntary contraction, the relaxation capacity of the PFM is also recorded. To represent the overall muscle structure [57], the strength and relaxation status of the PFM was assessed symmetrically on all PFM quadrants (approx. 5:00/7:00/3:00/9:00 and 11:30/00:30). During vaginal palpation, the cough test was again used to examine involuntary PFM reflex contraction, descent problems and cystoceles/rectoceles [57]. In cases of functional limitations of the PFM, e.g., lack of tPMFC-A, MOS < 3 and/or occurring PFDs, the participants were referred for gynecological and, if necessary, physiotherapeutic care.

## 3. Results

### 3.1. Study Population

**Figure 1:**
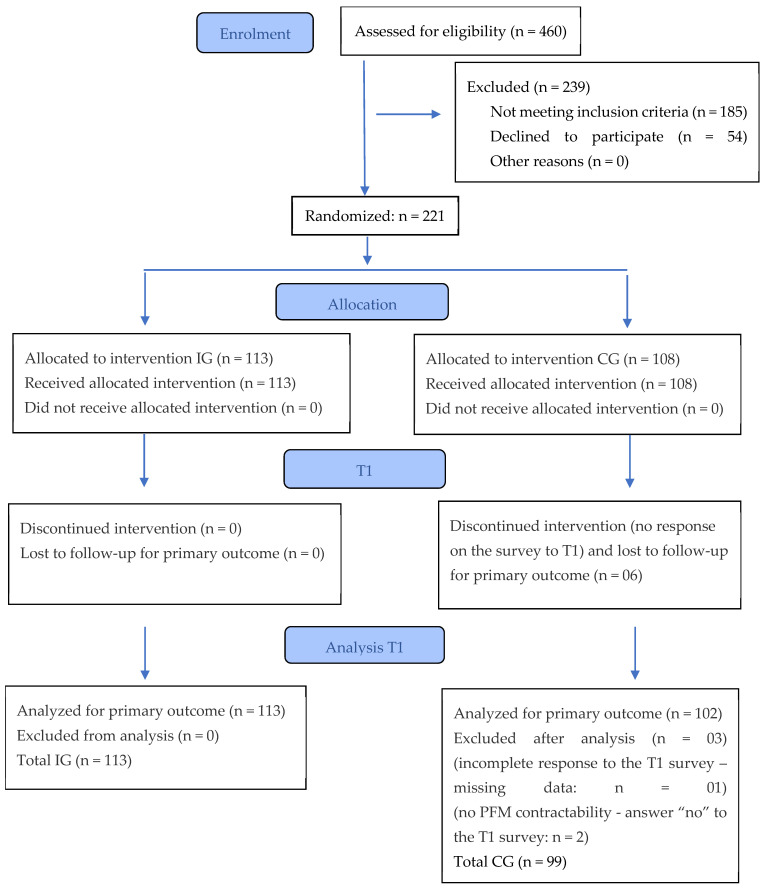
Illustration of the study phases of randomization and participation in the intervention and control group at T1, based on Hopewell et al. (2025) [60].

A total of 221 women was recruited for the study (Figure 1).

### 3.2. Characteristics and Risk Burden

The population of study participants in the IG (*n* = 113) (Figure 1) included a total of 69 nulliparous, 40 primiparous, and four biparous women. The characteristics and risk burden of the study participants shown are based on the first and sixth domains of the *GPFQppw* [40] and are shown in Table 1. In the survey, participants indicated a low overall risk burden for the item on self-assessed tPFMC-A.

### 3.3. Effect of Structured Teaching

**Figure 2:**
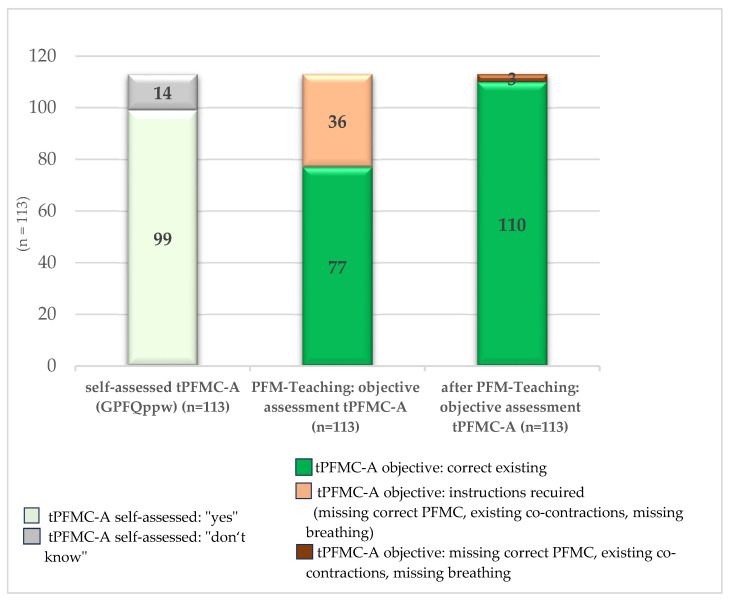
Results of the influence of teaching on the tPFMC-A: T1.

The theoretical and practical teaching on various aspects of PFM health described above was followed by an assessment of the targeted, correct tPFMC-A. Initially, over 87% (*n* = 99) of the pregnant women in the survey stated in a self-assessment that they were able to tense their PFMs in a targeted manner. Objective assessment by visual inspection showed that 68% (*n* = 77) actually demonstrated correct tPFMC-A. The main causes of incorrect or insufficient tPFMC-A were co-contractions of other muscle groups (gluteal muscles, hip adductors, rectus abdominis muscle), lack of targeted tension, and disturbed breathing flow. After specific instruction with verbal feedback (PFM teaching), 97% (*n* = 110) of the participants performed correct tPFMC-A without deficits (Figure 2).

Of the 36 participants who required and received instruction on correct and unrestricted tPFMC-A, six participants (16.7%) demonstrated a lack of correct tPFMC-A in combination with co-contractions and a lack of breath flow. Another 30 participants (83.3%) showed restricted tPFMC-A. The main deficit in 20 women (55.6%) was co-contractions. Seven (19.4%) participants showed co-contractions in combination with impaired breath flow. Three (8.3%) participants showed only impaired breath flow, which affected the tPFMC-A.

Within the group that required corrective guidance, there were 19 (16.8%) nulliparous women and a total of 17 (15.0%) primiparous and biparous women. In the *GPFQppw* survey, affected nulliparous women predominantly rated self-assessed ability to perform tPFM-A as “yes”. Only three (15.8%) responded with “I don’t know”. Affected primiparous and multiparous women also rated their self-assessed ability to perform tPFMC-A predominantly positively. Only one of them (5.9%) answered “I don’t know”. Overall, the participants overestimated their self-assessed tPFMC-A compared to their objectively tested actual contraction ability.

After instruction and verbal feedback, 97.3% (*n* = 110) of the participants demonstrated correct tPFMC-A. Three participants (2.7%) did not demonstrate correct tPFMC-A despite repeated instruction and feedback, with manifest co-contractions. At the same time, they rated their self-assessed ability to perform tPFMC-A in the *GPFQppw* as “yes” in each case. This subgroup consisted of one nullipara, one primipara, and one bipara, each of whom had a pathological Oxford score of 1 (MOS) at T1 of the study. In these cases, further gynecological or physiotherapeutic diagnostics were performed.

### 3.4. Functional Diagnostics: Vaginal Palpation and PFM Strength (MOS)

Vaginal palpation to assess PFM strength yielded the following distribution on the modified six-point Oxford Grading Scale (MOS): MOS 0 = 0 participants; 1 = 3 (2.6%); 2 = 0; 3 = 68 (60.2%); 4 = 40 (35.4%); 5 = 2 (1.8%). The median MOS was 3 (IQR 3–4). Thus, the majority of participants showed moderate-to-good PFM strength.

When PFM strength is differentiated according to parity, a statistically significant stronger PFM strength is observed in participating nulliparous women (*n* = 69) (*p* < 0.001). This has a median of MOS 4 (IQR 3–4), compared to participating primiparous women (*n* = 40), whose MOS has a median of 3 (IQR 3–3). Biparous women (*n* = 4) show a median PFM strength of MOS 3.5 (IQR 3–4), but this is not very meaningful given the very small number of cases.

### 3.5. Self-Reported PFM Health (GPFQppw)

While the self-assessment of the tPFMC-A presented in the previous chapter is based on the *GPFQppw* question “Can you consciously tense your pelvic floor muscles?”, at this point, the self-reported PFD risk symptoms are derived from the different four functional areas of the *GPFQppw* and reflect the general PFD symptom status. In principle, all participants who scored ≥ 1 (“sometimes/a little”) on one or more items in the respective domain were defined as symptomatic according to Metz et al. (2017) [39]. The following criteria correspond to the defined methodological specification for the classification of PFD symptom severity and were applied using the *GPFQppw* [39,40]:

− Urinary incontinence (UI): Positive responses to at least one of the questions on nocturnal enuresis, urge incontinence or stress incontinence.
− Anal incontinence (AI): Positive responses to questions on involuntary loss of wind or stool or stool smearing.
− Descensus symptoms: Positive answers to questions about foreign body sensation or a feeling of descensus in the vagina or uterus.
− Sexual symptoms: Positive answers to questions about vaginal lubrication, changes in vaginal sensitivity, and vaginal tightness and/or wideness.

The evaluation of the *GPFQppw* showed that participants exhibited various self-reported symptoms of PFDs within the four functional domains (bladder, bowel, continence and sexual function) (Table 2).

Stress incontinence was the most common symptom of urinary incontinence. The main symptom of anal incontinence was flatulence. The most common complaint associated with descent was a feeling of sinking. The most common sexual symptom was dyspareunia (Table 3).

After analyzing the self-reported PFD symptoms according to parity (Table 4), clear differences in the distribution of urinary incontinence and descent symptoms became apparent. Primiparae and biparae reported significantly more frequent symptoms of urinary incontinence (79.5% vs. 31.9%) and descent complaints (54.5% vs. 15.9%) than nulliparas (*p* < 0.001). In contrast, differences with regard to anal incontinence or sexual symptoms were not significant.

In summary, the results show that PFDs frequently occur during pregnancy. Pregnant women who have already given birth, in particular, show a significantly increased incidence of urinary incontinence and descensus symptoms.

## 4. Discussion

This study examines the PFM health of pregnant women as part of the “pelviTrust” Trial. The focus is on analyzing measurement point T1, which provides insight into the functional and subjectively perceived status of the PFM in early pregnancy.

Before discussing the central question of the relationship between subjective perception and objective PFM contraction ability, we will first consider the findings on the general prevalence of PFDs. These findings provide the necessary context for classifying the functional significance and preventive approach of PFM teaching. Although the results should be interpreted with caution due to the study design and the restriction to a single early-pregnancy time point, they indicate that symptoms of pelvic floor dysfunction frequently occur during pregnancy in our study population. This finding is confirmed by various international reviews [3,13,61].

More than half of all participants in our study reported symptoms of stress incontinence and flatulence as signs of anal incontinence at T1. The data on stress incontinence is consistent with the findings of Mossdorff–Steinhauser et al. (2021), who describe an average prevalence of urinary incontinence during pregnancy of 41% in their review of 88,305 pregnant women [2]. The most common form of urinary incontinence is stress incontinence, accounting for up to 63% of cases. The incidence of new urinary incontinence symptoms in nulliparous women is between 16.4% and 21.7% in early pregnancy and up to 63.2% at the end of pregnancy. In this study, stress incontinence is the most common form of newly occurring urinary incontinence, accounting for 70% of cases [2].

The number of pregnant women reporting symptoms of anal incontinence at time point T1 appears high in a study population of 61.1% nulliparae. Studies show that multiparous women, especially those who have had vaginal surgical deliveries, are particularly affected by symptoms of anal incontinence [4,62]. However, in our study, those who are nulliparous who reported anal incontinence symptoms mainly exhibited the symptoms of flatulence (63.8%). The high prevalence could, however, also be due to the broad definition of fecal incontinence in the *GPFQppw*, which includes involuntary gas loss. This symptom pattern may reflect physiological changes typical of early pregnancy rather than established anal sphincter dysfunction. Progesterone-mediated relaxation of smooth muscle and the mechanical action of the growing uterus can lead to increased gas loss in early pregnancy [6]. A longitudinal follow-up is needed to clarify whether these early-pregnancy symptoms persist postpartum and whether they correspond to true anal incontinence. Nevertheless, Solans–Domènech et al. (2010) show similar results in their study: in a study population of 1128 healthy, nulliparous women, flatulence is the main symptom of anal incontinence in up to 90% of cases throughout the entire pregnancy [63].

Descensus symptoms occurred in a quarter of the IG at T1. In her longitudinal study, Metz (2015) showed that 2.6% of the initial 233 nulliparous study participants exhibited pre-pregnancy descensus symptoms, which increased to 25.0% in the third trimester of pregnancy [64]. Dyspareunia as a sign of sexual dysfunction was reported by more than half of participants at T1 in our study (55.8%), a prevalence higher than that reported by Metz (2015), which shows a more moderate prevalence in her study group: the symptom of dyspareunia increases from 19.9% pre-pregnancy to 31.8% in the third trimester of pregnancy [64]. These discrepancies may be related to methodological differences and the timing of assessment and should therefore be interpreted cautiously [64].

The extent to which the PFDs reported in our study population at T1 manifest themselves is to be studied. In their review, Hübner et al. (2022) illustrated that the prevalence of urinary and fecal incontinence increases during pregnancy and peaks at the time of delivery [3]. Six months postpartum, significantly fewer women show signs of urinary or fecal incontinence. Nevertheless, up to 38% of women are still affected by persistent urinary incontinence 20 years after given birth, and 6% by fecal incontinence [3]. Our data, however, do not allow conclusions regarding postpartum or long-term PFM outcomes.

Parity proved to be a key factor in the occurrence of PFD symptoms during pregnancy. Women who had already given birth showed significantly more symptoms of urinary incontinence and descent than nulliparous women. This result is consistent with studies that describe parity and vaginal delivery as major factors for PFDs, due to repeated mechanical stress, stretching of connective tissue and nerve strain [6,7,65]. Overall, our results from self-reported PFM health show that PFDs during pregnancy are not isolated cases, but a widespread phenomenon that requires structured preventive measures by healthcare providers.

A central finding of this study is the discrepancy between pregnant women’s subjective perception of their ability to contract the PFM; the study objectively assessed contraction ability. At T1, many participants assumed that they were able to activate their PFM correctly and without restriction, although the objective examination showed limited or incorrect contraction at T1.

To date, there has been limited research into the self-perception of targeted PFM contraction in pregnant women. In their study involving 300 pregnant women, Hilde et al. (2012) showed that initially 94% of pregnant women stated that they could tense their PFM, while 6% were unsure [66]. After verbal feedback on PFM contraction behaviour and subsequent measurement of PFM strength, 12 women (4%) were identified who did not perform PFM contraction correctly. The authors report that straining instead of PFM contraction was the main error [66]. In our study, 36 women (32%) showed initial incorrect or insufficient PFM contractility. The main errors were co-contractions of other muscle groups (gluteal muscles, hip adductors, rectus abdominis muscle) and disturbed breathing flow. After repeated instruction and verbal feedback, three participants (2.7%) did not demonstrate correct tPFMC-A. Our findings are also reflected in the Brazilian study by Uechi et al. (2020) with *n* = 82 non-pregnant women of different ages (mean age of 46.83) (±17.94) [67]. The study shows also interesting results: 98% of women believed they could voluntarily tense their PFM, but only 33% of the study participants had a correct self-perception of their PFM contraction compared to the assessment by the physiotherapist using vaginal palpation. These participants achieved an MOS score of at least 3 [67].

The current literature is divided on the ability of women to learn how to perform correct PFM contraction after receiving verbal instructions. Some studies show that verbal instructions are not sufficient to improve PFM contraction in women. Bump et al. (1991) [25] found that 25% of women still could not perform correct PFM contraction after standardized verbal instructions. De Andrade et al. (2018) found no significant improvement in maximum voluntary PFM contraction or contraction ability after teaching the “Knack” in healthy women [68]. Talasz et al. (2012) reported persistent deficits despite brief instruction [69]. The improvement in our cohort suggests that combining theoretical instruction with visual inspection, vaginal palpation and individualized feedback may be more effective than verbal instruction alone for improving targeted contraction ability in early pregnancy. The three participants in our study who were unable to achieve adequate tPFMC-A despite repeated instruction consistently showed pathological values on the MOS (1–2). These women were referred for gynecological investigations, as physiotherapy support may be indicated in cases of suspected PFD in order to improve PFM function during pregnancy [70,71].

The results of our study, in the given study population, suggest that structured instructions for PFM contraction and vaginal palpation could be effective methods for identifying women with ineffective or faulty PFM contraction. They also indicate that affected women require early and targeted (uro-)gynecological or physiotherapeutic diagnostics, as this is crucial for effective prevention, rehabilitation, and treatment within the context of early-pregnancy care.

Our intervention was conducted by a midwife. Previous research often relies on physiotherapist-led programmes [14,27,67,68,72,73]. The improvement rate observed in our study suggests that the teaching concept used may also be promising for midwives as a profession group in supporting PFM perception and contraction ability in early pregnancy. For midwifery practice, however, this would also mean that PFM education and ensuring correct PFM contraction should be systematically integrated into the setting of continuity prenatal midwifery care [74]. To this end, training concepts and curricula should be expanded and specified as part of the academization of midwifery. Although Germany currently lacks a national framework for standardized content to promote PFM health in women in the context of pregnancy and childbirth, as well as clearly defined responsibilities in relevant professional guidelines [24,75], midwives have the necessary proximity and expertise to guide women in perception, understanding of function and PFM-friendly behaviour. Measures such as conscious pre-contraction (“The Knack”) during exertion or economical movement patterns when lifting and carrying [50,51,52,53,54,58] can be specifically taught. Because our findings are limited to early-pregnancy time point, longitudinal analyses are required to determine whether the observed functional improvements persist postpartum or translate into longer-term pelvic floor outcomes.

## 5. Limitations

The “pelviTrust” study is a pilot study for further research in the field of midwifery science. Potential limitations arising from the pragmatic approach of the study should be taken into account, particularly the following: Although recruitment was expanded beyond the Perinatal Centre of the University Hospital of Tuebingen, the recruitment period had to be ended before the planned sample size was reached. This could limit the statistical significance of the study results. To assess PFM health in pregnant women in the Tuebingen area, nulli-, primi- and biparous women were included in our study. Although the study population reflects the actual population, the comparability of the groups and their implicit transfer, the results of our study are made more difficult due to limitations. Although the study population examined attempts to reflect the PFM health of pregnant women, the comparability and transferability of our study are limited:

The limited exclusion criteria of the study, especially the varying burden of pre-existing PFD on participants with different parity, as well as the existing sample size of the study population, represent potential, unrecorded confounding factors that influence causal conclusions, which may distort results, having a negative effect on internal validity.

Although the participants were enrolled in the study by two people—the midwife who conducted the study and intervention and the associated principal investigator—the randomization process was carried out by the same midwife who also carried out the intervention. Randomization followed a standardized urn procedure; nevertheless, the absence of an independent second person introduces potential for unintentional selection bias. This form of randomization could unconsciously lead to participants who appear particularly motivated being assigned to the intervention group. As a result, inadequate allocation concealment can lead to an overestimation of the intervention and thus to minimized internal validity. Future studies should delegate randomization to an independent person in order to strengthen allocation concealment.

Due to its design, the study is unblinded, both for the participants and for the midwife conducting the study. Therefore, the influence of possible performance bias on the study result cannot be ruled out. Although it can be assumed that all study participants are motivated to improve their PFM health simply by participating in the study, the current study design offers the possibility of performance bias. The intervention group may be more motivated to engage in PFM training than the control group, which only completes questionnaires. Participants in the intervention group may rate their tPFMC-A score particularly positively later in the study due to the specific sensitization, as compared to the control group.

Even though the fact that all examinations were carried out by a specially trained midwife throughout the “pelviTrust” study period, which should help ensure intra-rater reliability, the single-examiner approach inherently limits blinding and may introduce detection bias. The involvement of a second reviewer would improve validity and objectivity. For our study, internal validity would have been strengthened in particular by a second person collecting the findings. The possible confounding variables of detection and performance bias during the assessment of the Oxford score could thus have been demonstrated by the inter-rater reliability shown.

A potential follow-up study would require a larger sample size to account for the different characteristics of the parities. A multicentre study design should also be considered to ensure the generalizability of the study results to the general population. In conclusion, we nevertheless believe that in our study, through standardized diagnostics, the use of validated instruments and clearly defined intervention components can provide a solid basis for further research.

## 6. Conclusions

In summary, this study shows that many pregnant women in our study population overestimate their self-assessed tPFMC-A. Based on a cautious interpretation of our exploratory approach study, these findings suggest improved functional control and increased awareness of their PFM among pregnant women, which could represent a promising model for preventive prenatal care. In addition, pregnant women with PFDs were identified and referred for early treatment. Pregnancy is considered a “teachable moment” for women—they are particularly receptive to health information and motivated to participate in preventive measures [76,77]. Midwives can use this receptiveness to provide early education, recognize pregnancy-related PFD symptoms, offer preventive training and, if necessary, refer pregnant women for further diagnosis. Structured PFM teaching should therefore be integrated into midwifery practice as an integral part of antenatal care.

From the perspective of midwifery science, this requires midwives to have, on the one hand, in-depth knowledge of PFM health and potential risks that may affect it. In addition, application-specific knowledge is necessary, such as how to perform a vaginal palpation assessment and how to use screening questionnaires to evaluate PFM health. In our opinion, these skills should be included in midwifery training curricula and in continued professional development in order to contribute to the promotion of pregnant women’s PFM health.

## Figures and Tables

**Table 1:** IG participant’s characteristics and self-reported risk burden (*n* = 113).

Characteristics and Self-Reported Risk Burden in GPFQppw	IG (*n* = 113)
Age (years)	32.4 ± 3.3
Highest educational qualification:	
College or university	96 (85.0%)
High school, secondary school and other	17 (15.0%)
Parity	
0	69 (61.1%)
1	40 (35.4%)
2	4 (3.5%)
Gestational age (week)	20 (IQR 19–21)
Body mass index (BMI) (pre-pregnancy)	22.6 (IQR 20.7–24.7)
Body mass index (BMI) (18th–22nd week of pregnancy)	24.1 (IQR 22.1–26.0)
Nicotine abuse	15 (13.3%)
Previous vaginal births	34 (30.1%)
Previous vaginal surgical births (vacuum extraction or forceps)	6 (5.3%)
Previous surgical birth (sectio caesarea)	4 (3.5%)
Previous macrosomia child	6 (5.3%)
Previous obstetric anal sphincter injuries (OASI III° or IV°)	10 (8.8%)
Familial risk burden of urinary incontinence	29 (25.7%)
Targeted PFM contraction ability (tPFMC-A)	
Yes	99 (87.6%)
Do not know	14 (12.4%)
No	0 (0%)

Values are presented as mean ± standard deviation, *n* (%) or median (inter-quartile-range (IQR)).

**Table 2:** Overall prevalence of existing PFD symptoms at T1.

T1: Self-Reported PFD Symptoms (GPFQppw)	IG (*n* = 113)
UI symptoms	57 (50.4%)
AI symptoms	76 (67.3%)
Descensus symptoms	35 (31.0%)
Sexual symptoms	84 (74.3%)

Values are *n* (%), unless otherwise specified.

**Table 3:** Prevalence of existing PFD and their subcategories at T1.

T1: Self-Reported PFD Symptoms by Subcategories (GPFQppw)	IG (*n* = 113)
UI symptoms:	
Urge urinary incontinence	24 (21.2%)
Stress urinary incontinence	54 (47.8%)
Mixed urinary incontinence	21 (18.6%)
Enuresis nocturna	3 (2.7%)
AI symptoms:	
Fecal incontinence	2 (1.8%)
Stool smearing	20 (17.7%)
Flatulence	66 (58.4%)
Descensus symptoms:	
Vaginal foreign body sensation	10 (8.8%)
Sinking feeling	28 (24.8%)
Feeling of sinking when moving	27 (23.9%)
Sexual symptoms:	
Lack of moisture	15 (13.3%)
Vaginal sensory reduction	43 (38.1%)
Vaginal width	24 (21.2%)
Vaginal tightness	29 (25.7%)
Dyspareunia	63 (55.8%)

Values are *n* (%), unless otherwise specified.

**Table 4:** Prevalences of PFD symptoms differed in parity at T1.

T1: Self-Reported PFD Symptoms (GPFQppw)	IG Study Population (*n* = 113)		
	0-Para (*n* = 69)	>0-Para (*n* = 44)	*p*-value
UI symptoms	22 (31.9%)	35 (79.5%)	<0.001 *
AI symptoms	44 (63.8%)	44 (72.7%)	0.322
Descensus symptoms	11 (15.9%)	24 (54.5%)	<0.001 *
Sexual symptoms	50 (72.5%)	34 (77.3%)	0.568

Values are *n* (%), unless otherwise specified; Chi-square test was used; * *p* < 0.05 indicates statistical significance.

## Data Availability

The datasets presented in this article are not readily available because the data are part of an ongoing study. The data will be disclosed after the study has been published in full (via DRKS).
